# Wild Type Mesenchymal Cells Contribute to the Lung Pathology of Lymphangioleiomyomatosis

**DOI:** 10.1371/journal.pone.0126025

**Published:** 2015-05-15

**Authors:** Debbie Clements, Arundhati Dongre, Vera P. Krymskaya, Simon R. Johnson

**Affiliations:** 1 Division of Respiratory Medicine, University of Nottingham, Nottingham, United Kingdom; 2 Pulmonary, Allergy, & Critical Care Division University of Pennsylvania, Perelman School of Medicine, Philadelphia, PA, United States of America; Helmholtz Zentrum München, GERMANY

## Abstract

Lymphangioleiomyomatosis (LAM) is a rare disease leading to lungs cysts and progressive respiratory failure. Cells of unknown origin accumulate in the lungs forming nodules and eventually resulting in lung cysts. These LAM cells are described as clonal with bi-allelic mutations in TSC-2 resulting in constitutive mTOR activation. However LAM nodules are heterogeneous structures containing cells of different phenotypes; we investigated whether recruited wild type cells were also present alongside mutation bearing cells. Cells were isolated from LAM lung tissue, cultured and characterised using microscopy, immunocytochemistry and western blotting. Fibroblast-like cells were identified in lung tissue using immunohistochemical markers. Fibroblast chemotaxis toward LAM cells was examined using migration assays and 3D cell culture. Fibroblast-like cells were obtained from LAM lungs: these cells had fibroblast-like morphology, actin stress fibres, full length tuberin protein and suppressible ribosomal protein S6 activity suggesting functional TSC-1/2 protein. Fibroblast Activation Protein, Fibroblast Specific Protein/S100A4 and Fibroblast Surface Protein all stained subsets of cells within LAM nodules from multiple donors. In a mouse model of LAM, tuberin positive host derived cells were also present within lung nodules of xenografted TSC-2 null cells. In vitro, LAM 621-101 cells and fibroblasts formed spontaneous aggregates over three days in 3D co-cultures. Fibroblast chemotaxis was enhanced two fold by LAM 621-101 conditioned medium (p=0.05), which was partially dependent upon LAM cell derived CXCL12. Further, LAM cell conditioned medium also halved fibroblast apoptosis under serum free conditions (p=0.03). Our findings suggest that LAM nodules contain a significant population of fibroblast-like cells. Analogous to cancer associated fibroblasts, these cells may provide a permissive environment for LAM cell growth and contribute to the lung pathology of LAM lung disease.

## Introduction

Lymphangioleiomyomatosis (LAM) is a rare and progressive multi-system disease affecting women, which leads to respiratory failure over a variable period of time[1]. LAM can occur sporadically, but is common in patients with tuberous sclerosis complex (TSC). Histological examination shows that a heterogeneous population of mesenchymal cells, termed LAM cells, infiltrate the lungs and lymphatics of these patients. Although women with LAM may develop lymphatic masses, chylous collections and the tumour angiomyolipoma, the main morbidity is caused by the lung disease [2]. Within the lung parenchyma, LAM cells form nodular aggregates and, probably due to the production of proteolytic enzymes [3, 4], damage lung tissue to form cysts, which gradually increase in number.

To date, understanding the pathology of the lung disease has focused on the LAM cell: a cell type with no known normal counterpart. These cells have been described as displaying markers of both smooth muscle lineage, including actin and desmin and those suggestive of neural crest development including glycoprotein 100 and the micropthalmia transcription factor (MITF)[5]. Although the normal precursor of the LAM cell is unknown, this ‘dual phenotype’ places the lesion in the perivascular epithelioid cell (PEC) group of neoplasms also including angiomyolipoma and clear cell tumour of the lung[6].

In the majority of cases examined, LAM cells and other PEComas harbour mutations in TSC-2 resulting in constitutive activation of the mechanistic (previously mammalian) target of rapamycin (mTOR)[7], a pivotal cellular kinase controlling growth, metabolism and autophagy[8]. Within the same patient, LAM cells isolated from multiple sites including the lungs, lymphatics, kidneys and those present in blood and other body fluids have identical TSC-2 mutations [9]; suggesting that LAM cells are clonal and capable of metastasising [10]. Despite this assumed clonal nature, it has been noted for many years that LAM nodules in the lungs are heterogeneous structures, containing cells with both epithelioid and spindle-like morphologies[11]. Antibodies recognising alpha smooth muscle actin and phosphorylated P70^S6 kinase^ appear to react with all of these different cell populations. However, antibodies targeting either melanoma proteins, such as HMB-45 (anti-gp100/Pmel17/PMEL) and PNL2, or anti-oestrogen receptor alpha detect a variable subpopulation of cells within nodules, which tend to have the epithelioid phenotype [11, 12]. Importantly the expression of CD9 and CD44v6, has been associated with bi-allelic inactivation of TSC-2 in circulating LAM cells, and these markers are expressed in only 20% of cells within nodules [13]. Although these findings could be explained by differentiation of cells into discreet populations within nodules: many groups have attempted unsuccessfully to culture pure populations of mutation bearing LAM cells from lung tissue. Furthermore, next generation sequencing of TSC-2 mutations in carefully microdissected LAM nodules suggests that significant numbers of non-mutation bearing cells are present [14, 15]. One model that would explain this cellular heterogeneity and lack of expression of markers of TSC-2 loss is the presence of non-mutation bearing wild type cells within LAM nodules.

We postulated that LAM cells recruit wild-type mesenchymal cells to LAM nodules and that the association of the two cell types is analogous to that seen in cancer, where tumour cells recruit host fibroblasts to form mixed colonies of cells. This provides benefits to tumour cells including the provision of nutrients specific to the neoplastic phenotype, protection from immune surveillance, promotion of angiogenesis, metastasis and the production of proteases[16–18]. We therefore examined LAM tissue for the presence of wild type, non-LAM cells by a range of methods, and we show that LAM nodules contain wild type fibroblast-like cells which are attracted to LAM cells, in part by production of the chemokine CXCL12, and that LAM cell/fibroblast aggregates protect LAM cells from stress-induced apoptosis.

## Methods

### LAM Related Cells and Tissue

Lung tissue was obtained from patients with LAM undergoing diagnostic biopsies or lung transplant procedures as part of clinical care, or from the National Disease Research Interchange (NDRI). Ethical approval for the use of LAM lung tissue was given by the University of Nottingham Research Ethics Committee, and written, informed consent was obtained from all patients.

### Cell Isolation and Culture

Lung tissue was enzymatically dissociated by incubation in 2mg/ml collagenase (Sigma, Poole, UK) in serum free phenol red free Dulbecco's Modified Eagle Medium: Nutrient Mixture F-12 (DME-F12, Life Technologies Ltd, Paisley, UK) medium for 2–24 hours at 37°C, with occasional agitation. Cells released were washed in DME-F12 medium containing 10% foetal bovine serum, plated in the same medium and cultured on tissue culture treated plastic at 37°C and 5% CO_2_. Adherent cells were seen after 24 hours. Cells were used between passages 1 and 3. 621-101 cells are a TSC2-null cell line derived from a renal angiomyolipoma of a LAM patient and were a gift from Elizabeth Henske [19]. These cells were maintained in DME-F12 with 10% FCS. Normal Human Lung Fibroblasts (NHLFs) were purchased from Lonza (Slough, UK) and were maintained in DME-F12 with 10% FCS.

### Immunohistochemistry and Immunofluorescence

Paraffin sections were dewaxed in Histoclear (R.A. Lamb, Fisher Scientific, Loughborough, UK) and then rehydrated through a graded ethanol series. Where required, antigen retrieval was carried out in Dako Target Retrieval Solution (Dako UK Ltd, Ely, UK) for 20 minutes in a steamer. Endogenous peroxidases were inactivated by incubation in 3% hydrogen peroxide in water for 10 minutes, then sections were blocked for 30 minutes in 2.5% horse serum (Vector Laboratories, Peterborough, UK) before incubation with primary antibody at 4°C overnight. Washes were carried out in PBS with 0.5% Tween added (PBS-T). Secondary antibody incubation was carried out for one hour at room temperature. For peroxidase-conjugated secondary antibodies chromogenic detection was carried out using ImmPact DAB (Vector Laboratories) followed by counterstaining with Mayer’s Hematoxylin. Samples were mounted in Vectamount (Vector Laboratories). Where fluorophore conjugated secondary antibodies were used, samples were counterstained with 4',6-diamidino-2-phenylindole (DAPI) and mounted in Fluorescence Mounting Medium (Dako).

For immunofluorescent detection of proteins in cultured cells, cells were grown on 22 x 22mm glass coverslips in DME-F12 with 10% FCS for 24 hours. The cells were then fixed in 4% formaldehyde for 20 minutes at room temperature, and permeabilised in 0.1% Triton x100 in PBS for 5 minutes at room temperature. Samples were blocked in 2.5% horse serum, then incubated with primary antibodies overnight at 4°C. Fluorophore conjugated secondary antibodies were added for one hour at room temperature in the dark. Samples were DAPI stained if required, and mounted in Fluorescent Mounting Medium (Dako UK Ltd, Ely, UK).

Primary antibodies used were: rabbit anti-S100A4 ([EPR2761(2)], 1:100, Abcam, Cambridge, UK); rabbit anti-gp100 (ab137078, Abcam), anti-Fibroblast Activation Protein alpha antibody (ab53066, 1:100 Abcam); rabbit anti-E cadherin antibody (ab15148, Abcam); anti-Fibroblast Surface Protein (1B10, 1:100, Sigma); monoclonal Anti-α-Smooth Muscle Actin—Cy3 (Sigma C6198) 1:200; anti-Fibroblasts Antibody, clone TE-7 (Merck Millipore, Watford, UK); anti-Melanoma Associated Antigen PNL2 (Zytomed, Berlin, Germany); Anti-S100A4 antibody [NJ4F3] (ab68124, Abcam,); anti-tuberin (C-20, Santa Cruz Biotechnology Inc., Insight Biotechnology, Wembley, UK). Secondary antibodies were: Vector ImmPress HRP anti-Mouse and anti-Rabbit (Vector Laboratories, Peterborough, UK), Alexa Fluor 488 goat anti-mouse IgG antibody (Fisher Scientific, Loughborough, UK), Alexa Fluor 594 goat anti-rabbit IgG antibody (Fisher Scientific).

### Western blotting

Fibroblasts were grown to 50% confluence in 6 well plates in DME-F12 medium with 10% foetal bovine serum. Cells were serum starved where required by replacing this medium with serum-free DME-F12 for 24 hours, then replacing this with fresh serum-free DME-F12 for a further 24 hours. For rapamycin inhibition of S6 phosphorylation, medium was again replaced with fresh serum-free DME-F12 then 10nM rapamycin (InSolution Rapamycin, Merck Millipore, Watford, UK) was added 30 minutes prior to 10% foetal bovine serum. Cells were incubated for a further 24 hours then were lysed in 200ul per well of 2× SDS protein loading buffer containing β-mercaptoethanol. Samples were heat-treated at 94°C for 5 min and then subjected to SDS-PAGE on 10% acrylamide gels (Bio-rad Mini-Protean TGX, Bio-rad Hemel Hempstead, UK). Proteins were transferred to polyvinylidene difluoride (PVDF) membrane (GE Life Sciences, Little Chalfont, UK), which was blocked in PBS containing 0.1% Tween 20 and 5% dried milk powder. Membranes were incubated overnight at 4°C with primary antibodies diluted in blocking solution, then for an hour at room temperature in secondary antibodies. Signal was visualized by enhanced chemiluminescence (ECL, GE Life Sciences). Antibodies used were: anti-tuberin C-20 (Santa Cruz Biotechnology Inc), rabbit monoclonal anti phospho-S6 Ribosomal Protein (Ser235/236) XP (Cell Signaling Technology, New England Biolabs, Hitchin, UK), anti-S6 Ribosomal Protein (Cell Signaling) all at 1:1000 dilution. Secondary antibodies were horseradish peroxidase (HRP)-conjugated goat anti-mouse and goat anti-rabbit IgG (Sigma, Poole, UK) used at 1:10 000 dilution.

### Migration assays

Migration assays were performed essentially as described [20] using a Boyden chamber Transwell assay. Polycarbonate membrane Transwell inserts (8 μM pore size, Corning Life Sciences) were coated with 10μg/ml bovine collagen solution (Sigma) for one hour at room temperature prior to adding cells. Fibroblasts were resuspended in serum-free DME-F12 medium at a cell density of 10^6^ cells per ml. 10^5^ cells (100 μl) were then added to the upper chamber and underwent directional migration for 16–18 h towards 500 μl conditioned or control medium, or control medium with added recombinant CXCL12 (Peprotech, London, UK), in the lower chamber. Conditioned medium was harvested from 5x10^6^ 621–101 cells cultured in 15 mls of serum-free phenol red free DME-F12 for 24 hours, and filtered through a 22μm filter before use. Non-migrated cells were removed with a cotton swab then migrated cells fixed in 4% formaldehyde in PBS, stained with 1ug/ml DAPI in PBS and imaged on a Nikon Diaphot 300 inverted microscope (Nikon Instruments, Kingston upon Thames, UK) with epifluorescence under appropriate filters. Six fields per slide were imaged at ×20 magnification and migrated cells counted. Neither 621–101 conditioned medium nor recombinant CXCL12 at up to 100ng/ml caused fibroblast proliferation over the time scale of the experiment (not shown), suggesting the effects seen are due to migration rather than proliferation.

A bi-directional migration assay model was devised using metal-silicone inserts or ‘fences’ placed in 24-well tissue culture plates (Aix Scientific, Aachen, Germany). Prior to plating, NHLFs were stained with CellTracker Green CMFDA and 621–101 cells with CellTracker Orange CMTMR (Life Technologies Ltd, Paisley, UK) according to manufacturers’ instructions. In brief 10^6^ adherent cells were incubated in 1μM CellTracker Orange CMTMR (621–101 cells) or Green CMFDA (NHLFs) for 30 minutes at 37°C in serum free DME-F12. This was then replaced with fresh DME-F12 with no added dye. 1 x 10^4^ 621–101 cells were then plated in DME-F12 in the inner chamber of the fence with 4 x 10^4^ fibroblasts at the periphery, in the presence or absence of AMD3100. After 16 hours the fences were removed leaving a 4.6mm gap between the two cell populations. Medium was changed at 24 hour intervals and after 96 hours of culture in the absence of the fence, cells were fixed, DAPI stained and imaged on a Nikon Diaphot 300 inverted microscope with epifluorescence under appropriate filters. Cells were considered to have migrated if they had left the peripheral section and entered the area initially covered by the fence, or had reached the central reservoir.

### 3D cell culture

Fibroblasts and 621–101 cells were labelled with CellTracker dyes as above. A total of 10^5^ fibroblasts, 621–101 cells or 5:1 mixture of fibroblasts and 621–101 cells was embedded in 400μl of basement membrane extract (BME, Trevigen, AMS Biotechnology, Abingdon UK) in DME-F12 at a final concentration of 3.75mg/ml in 24 well plates. AMD3100 was included at 200 μg/ml where indicated. After an initial 24 hours incubation, gels were overlaid with 300μl DME-F12 with or without 200μg/ml AMD3100. This medium was replaced at 24h intervals.

Cells within the gels were imaged at 24 hour intervals, for up to 7 days, using a spinning disk confocal microscope (Improvision, Perkin Elmer UK).

### LAM Animal Model System

A mouse model of LAM was generated by Goncharova *et al*. as described previously [21]. In brief, six to eight week old female NCr athymic nu/nu mice (NCRNU-M, Taconic) were injected subcutaneously in both flanks with 5×10^6^ TSC2-null mouse kidney epithelial cells. Cells from resulting tumours were cultured for 2 days then 10^6^ of these cells were injected into the tail vein of 8-week-old NCRNU-M athymic nude mice. After 20 days these mice had developed TSC2-null lung lesions, alveolar airspace enlargement and parenchymal destruction, similar to human LAM. These animal procedures were approved by the University of Pennsylvania Institutional Animal Care and Use Committee.

For this study, lungs from these mice were paraffin embedded and sectioned for immunohistochemistry with an anti-tuberin antibody (anti-tuberin C-20, Santa Cruz Biotechnology Inc.) and anti phospho-S6 Ribosomal Protein (Ser235/236) (Cell Signaling Technology).

### Quantification of secreted CXCL12

CXCL12 levels were quantified in 621–101 cell serum free medium after 24 hours incubation using the Human SDF-1alpha Mini ELISA Development Kit (900-M92, Peprotech UK) according to manufacturer’s instructions.

### Apoptosis assay

1x10^4^ 621–101 cells/well were plated in 24 well plates, and serum depleted, or treated with NHLF conditioned serum free medium for 24 hours. Cells were fixed in 4% formaldehyde and the DNA fragmentation detected by TUNEL (Terminal deoxynucleotidyl transferase dUTP nick end labelling) using the Fluorescein In Situ Cell Death Detection Kit (Roche Diagnostics Limited, Burgess Hill, UK) according to manufacturer’s instructions. Cells were counterstained with 1ug/ml DAPI in PBS, imaged on a Nikon Diaphot 300 inverted microscope with epifluorescence under appropriate filters and the number of TUNEL (FITC) positive cells expressed as a percentage of the total number of DAPI stained nuclei.

### Statistical Analyses

Statistical analysis was performed using Graphpad Prism 6 software (Graphpad, La Jolla, USA). Paired experiments were analyzed by *t*-test and multiple comparisons by two-way ANOVA with Dunnett's or Bonferroni correction with a *P* value of <0.05 regarded as significant.

## Results

### Fibroblast-like cells are present in LAM lung nodules

**Fig 1:**
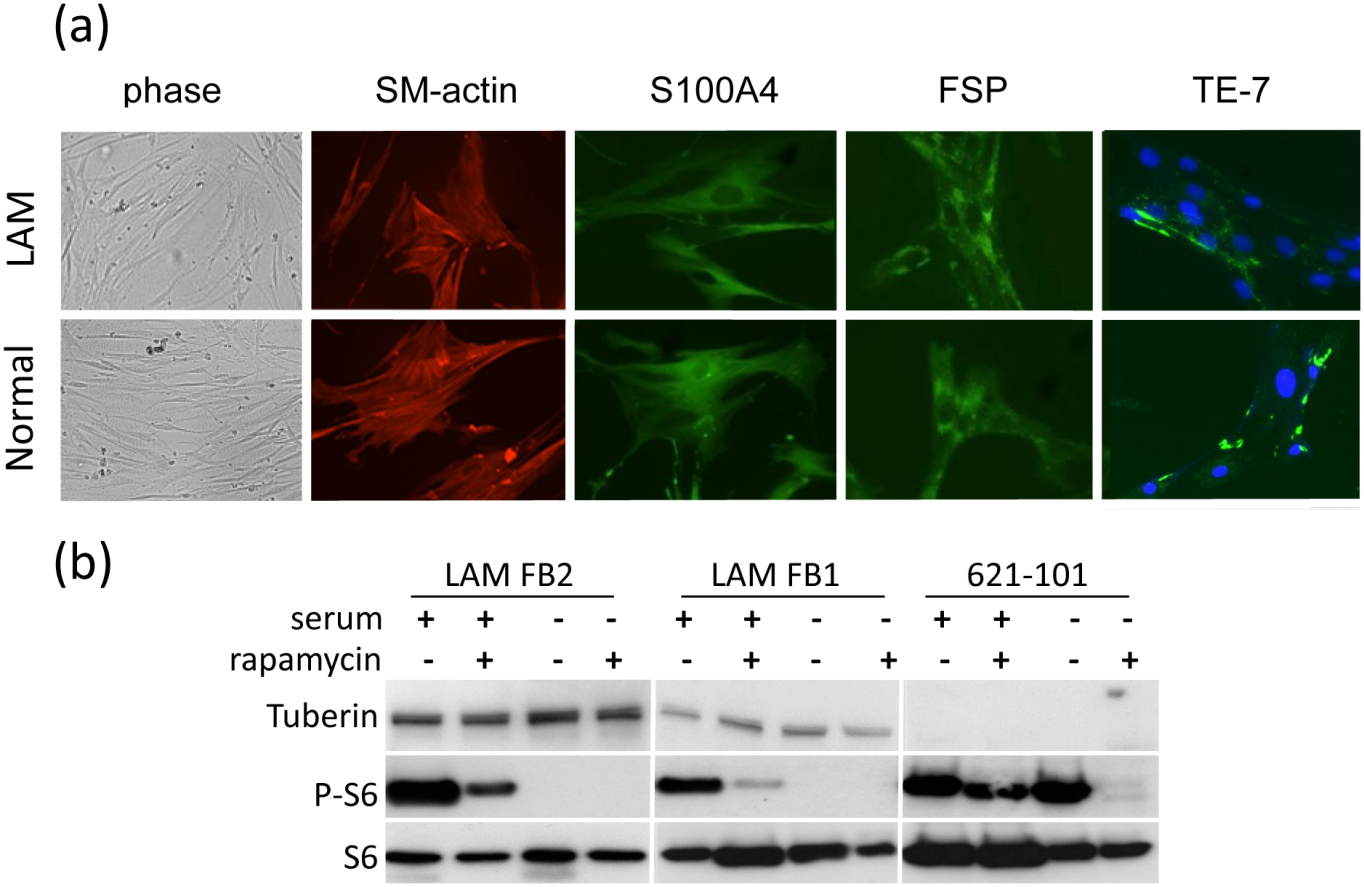
Characteristics of wild-type fibroblast-like cells obtained from LAM lungs. (a) Explant culture of lung tissue from patients with LAM yields a fibroblast-like population with spindle shaped morphology under phase contrast microscopy (phase) and immunoreactivity to anti-alpha Smooth Muscle Actin, and fibroblast markers FSP, S100A4 and TE-7 (these cells are also DAPI stained for clarity). (b) Western blot of lysates of fibroblast like-cells from two donors with LAM compared with TSC-2 mutation bearing 621–101 cells. Unlike 621–101 cells fibroblast-like cells have full length tuberin, and suppression of phospho-S6 in the absence of serum.

Lung tissue from three patients with LAM was treated with collagenase followed by culture in standard basal medium with 10% serum. Initial culture of the derived cell population yielded both spindle shaped and epithelioid adherent cells. After 14 days homogeneous populations of adherent, proliferative fibroblast-like cells were derived from all donors (Fig 1).

These fibroblast-like cells were indistinguishable from both primary human lung fibroblasts and commercially available normal human lung fibroblasts in terms of morphology and expression of FSP, S100A4, TE-7 and alpha-Smooth Muscle Actin (Fig 1a). They were negative for E-cadherin and gp-100 expression (data not shown). To determine if these cells expressed functioning TSC-1/2 protein we examined tuberin expression and activation of the mTORC1 target, ribosomal protein S6 by western blotting. Full length tuberin protein was detectable in all fibroblast-like cells and serum withdrawal led to suppression of S6 phosphorylation. Whereas in LAM derived 621–101 cells tuberin protein was undetectable and S6 phosphorylation persisted in the absence of serum (Fig 1b). Treatment with the mTOR inhibitor rapamycin led to a partial inhibition of S6 phosphorylation in all cell types. Next generation sequencing was available for cells from one donor. No mutation in either TSC-1 or TSC-2, or copy number variation, could be detected (not shown).

**Fig 2:**
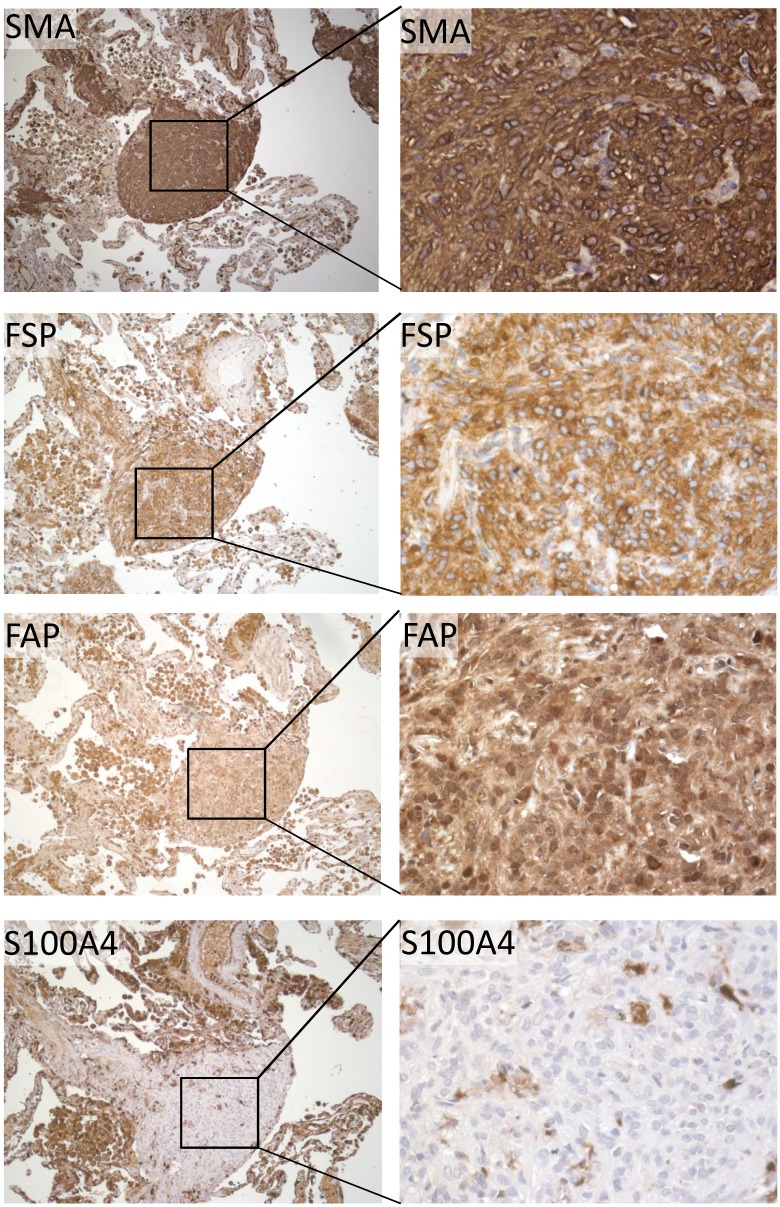
LAM nodules express fibroblast markers. LAM nodules react with antibodies against alpha-Smooth Muscle Actin, Fibroblast Surface Protein (FSP), Fibroblast Activation Protein (FAP) and S100A4. Left panels are x20 magnification and right are inset area at x40 taken from serial sections of a representative donor. Fibroblast markers display different expression patterns within LAM tissue, with anti-FSP reacting with 30–70% of cells within a nodule, anti-FAP detecting the majority of cells and S100A4 detecting only small nests of cells within nodules.

**Fig 3:**
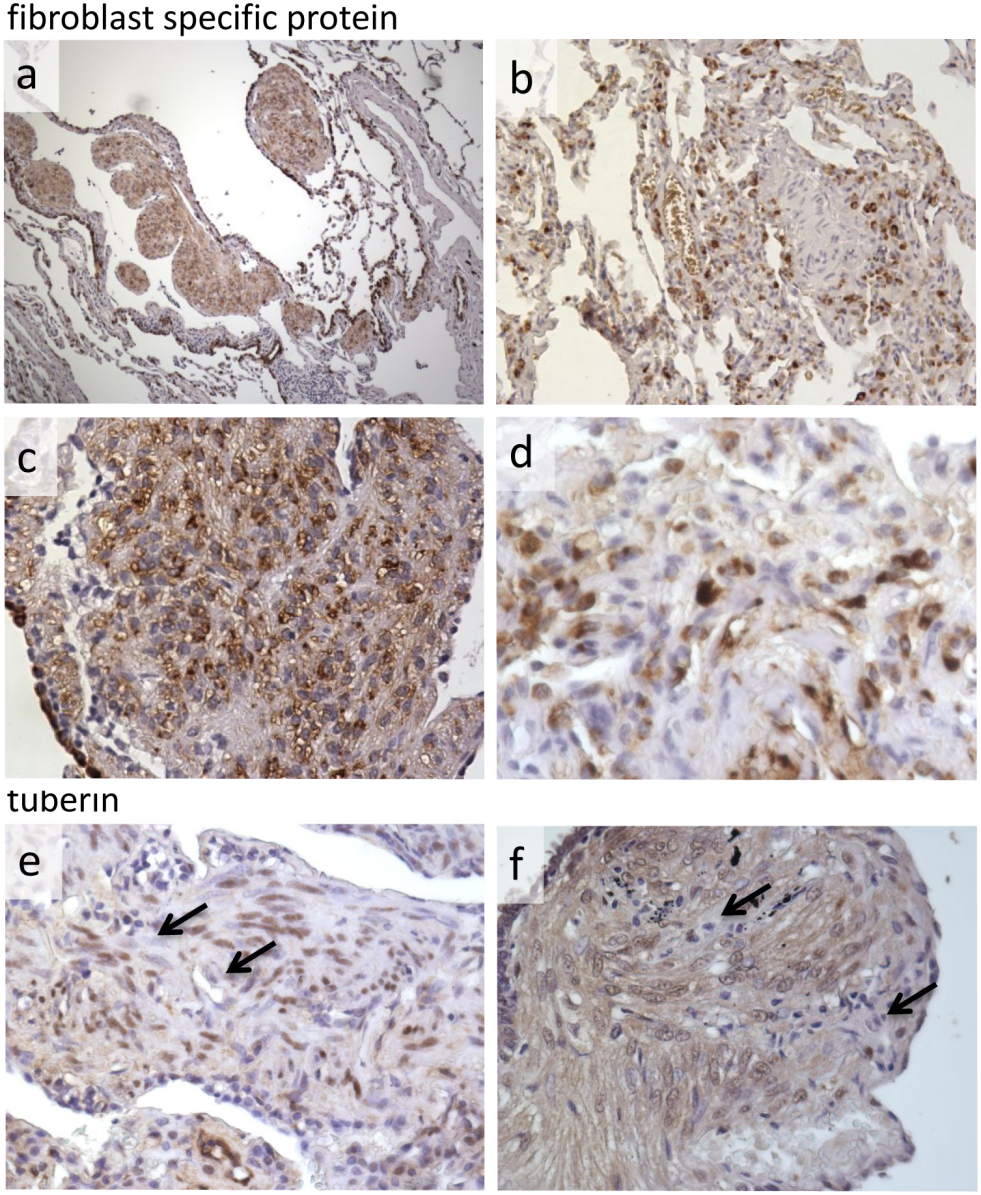
FSP is expressed by variable numbers of cells within LAM nodules. There is heterogeneity between different LAM lung donors in the number of anti-FSP immunoreactive cells in LAM nodules. Panels (a) and (b) at x10 magnification, (c) at x20, (d) at x40. (e) and (f) show anti-tuberin reactive and non-reactive cells (arrowed) within nodules from two donors, x20.

To investigate the likely origin of these cells within LAM lung tissue, we used immunohistochemistry with antibodies against established fibroblast markers to locate fibroblasts in situ. We found that sub-populations of cells within LAM nodules reacted with antibodies against Fibroblast Activation Protein (FAP), S100A4 and Fibroblast Surface Protein (FSP). Anti-FAP reacted with all stromal cells within LAM nodules and normal smooth muscle cells. Anti-S100A4 detected small nests of cells within nodules, normal smooth muscle cells, normal endothelial cells and alveolar epithelium. Anti-FSP reacted with a subset of cells within the stroma of nodules and strongly detected type II pneumocytes covering LAM nodules. Anti-FSP also detected type II pneumocytes but not type I pneumocytes from areas of normal lung, normal airway epithelium or vascular smooth muscle cells (Fig 2). FSP was present in variable proportions of cells in LAM nodules from different donors (Fig 3A–3D).

Previously it has been demonstrated that cells within LAM nodules are immunoreactive to anti-tuberin antibody [22]. Careful examination of LAM tissue sections treated with an anti-tuberin C terminus antibody showed that most cells did react to this antibody, as previously described, but also revealed a minority of cells which were not immunoreactive (Fig 3E and 3F).

**Fig 4:**
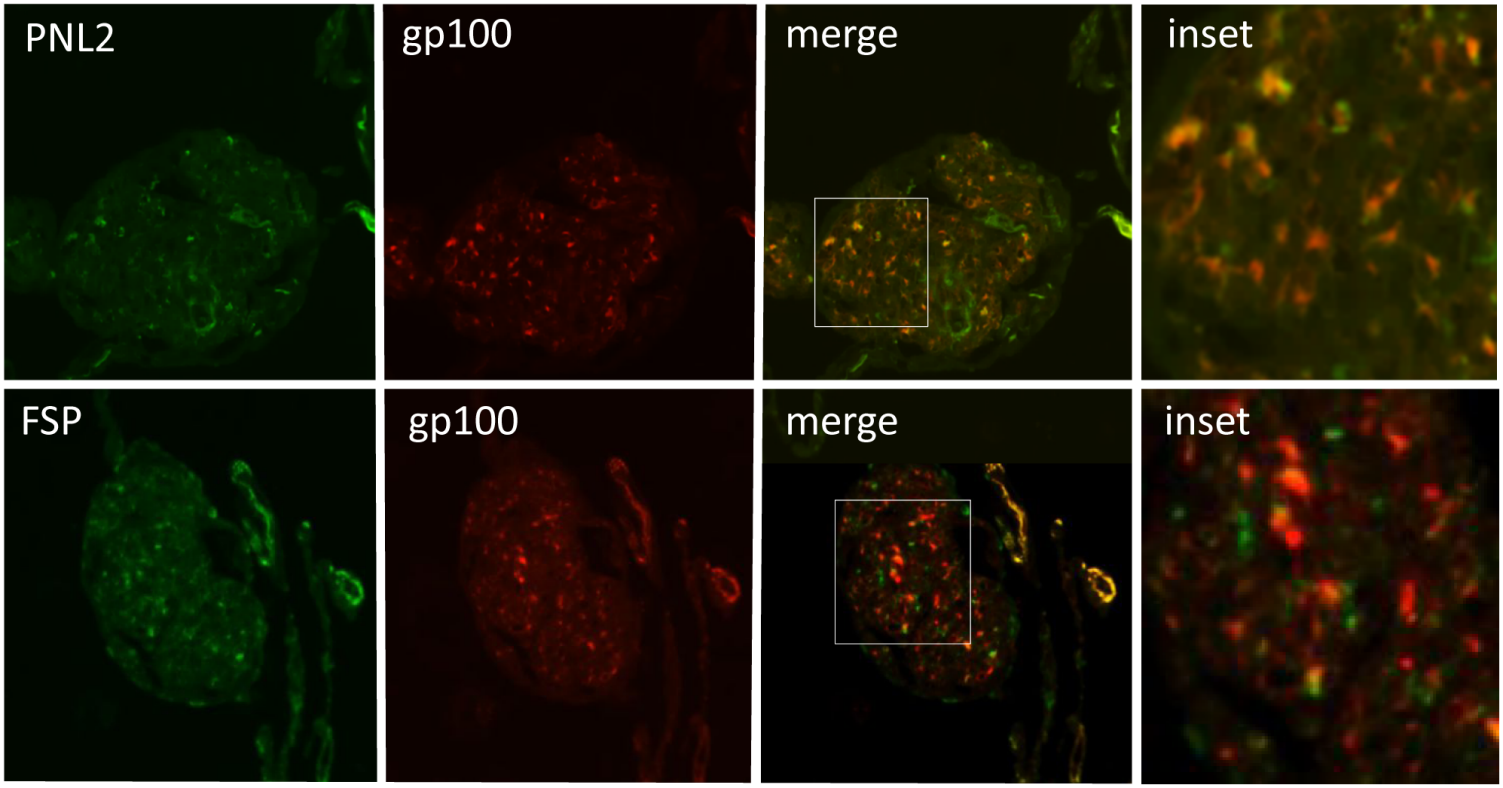
Fibroblast markers and melanoma markers are expressed in different cell populations within LAM nodules. LAM tissue subjected to immunofluorescence using melanoma marker antibodies PNL2 (green) and anti-gp100 (red) (the merged image shows cells which are positive for both markers and appear yellow/orange), and anti-gp100 (red) and the fibroblast marker anti-FSP (green), which do not overlap. Main images x10 magnification.

Antibodies against melanoma markers, such as anti-gp100/PMEL and PNL2 are used to identify LAM lesions. To determine if fibroblast and melanoma markers are expressed by distinct cell populations we performed double immunofluorescence with anti-gp100 in combination with the fibroblast marker anti-FSP. We had noted that our melanoma marker antibodies, PNL2 and anti-gp100, displayed a mostly overlapping pattern of immunoreactivity, indicating that they detect the same population of cells. However, the expression of FSP did not overlap that of gp100, suggesting that melanocyte and fibroblast markers are expressed by different populations of cells (Fig 4).

**Fig 5:**
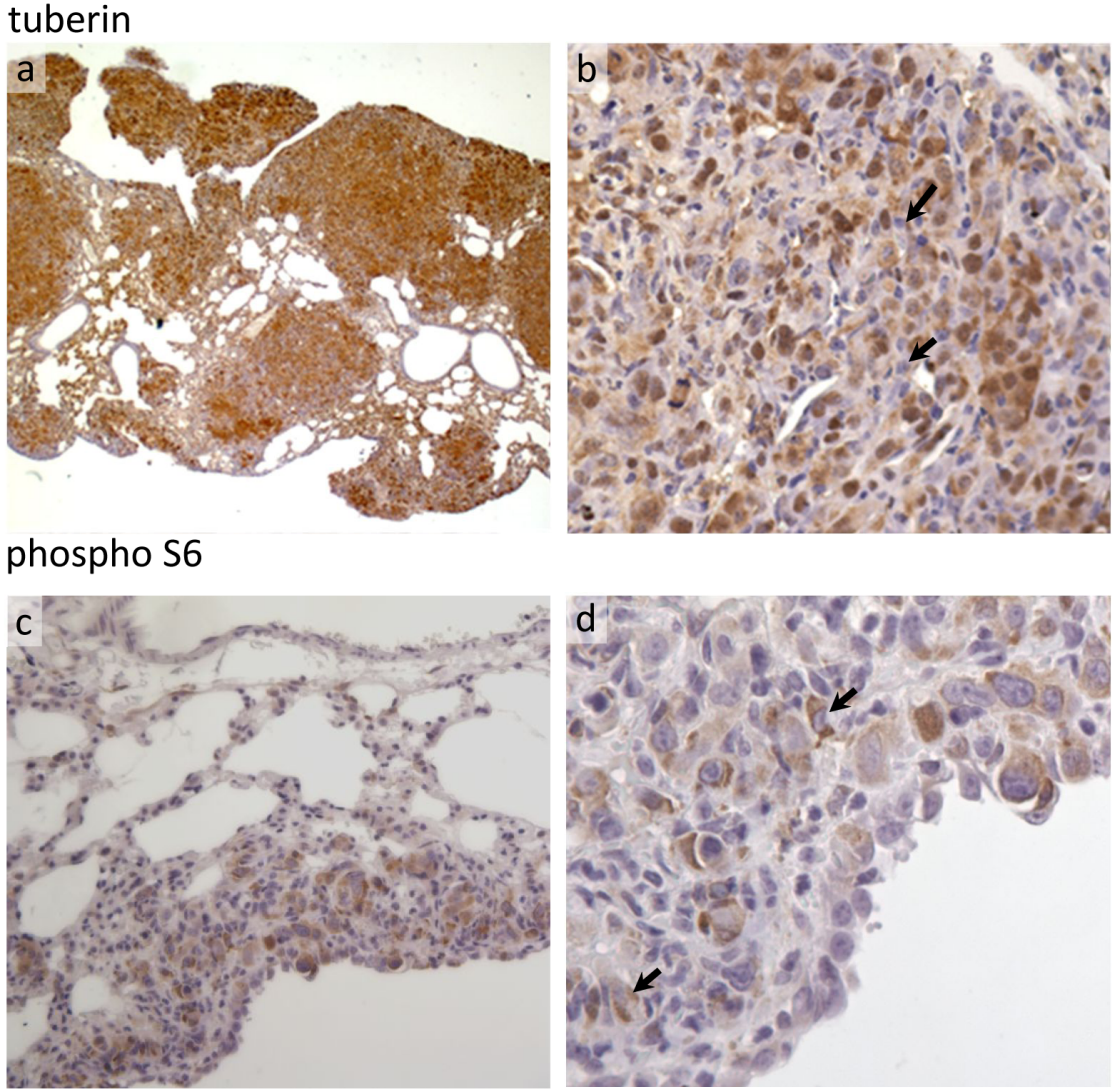
A mouse model of LAM also contains wild-type cells in lung nodules. NCRNU-M athymic nude mice received tail-vein injections of cells obtained from TSC-2^-/-^ mouse kidney epithelial tumours. Mice develop lung nodules and air space enlargement (a). Lung tissue was stained with an anti-tuberin antibody which shows a large number of tuberin positive (host) cells within nodules admixed with tuberin null (tumour) cells (b) examples highlighted with arrows, suggesting host cells have migrated toward the TSC2^-/-^ cells. The absence of phosphorylation of ribosomal protein S6 in many cells is also an indicator of functional tuberin activity in these cells.

To determine if TSC-2 null tumour forming cells were able to attract wild type cells in an alternative system, we examined the presence of recruited wild type cells in an animal model of LAM lung pathology. Goncharova *et al* [21] have developed a mouse LAM model which recapitulates many of the features of LAM in humans, including nodules of TSC-2 null cells in the lungs, enlargement of alveolar airspaces and destruction of lung parenchyma. These TSC-2 null cells do not express full length tuberin and do not react with an antibody which detects the C-terminus of tuberin protein (data not shown). However, within nodules there were large numbers of immunoreactive tuberin positive cells admixed with a smaller number of tuberin deficient cells (Fig 5). The phosphorylation status of ribosomal protein S6 can be used as a surrogate marker for mTOR dysregulation, with phosphorylation an indicator of uncontrolled mTOR activity. Immunohistochemistry using an antibody against the phosphorylated form of S6 revealed the presence of cells without S6 phosphorylation, suggesting functional tuberin expression (Fig 5).

### Recruitment of fibroblast-like cells to LAM nodules

**Fig 6:**
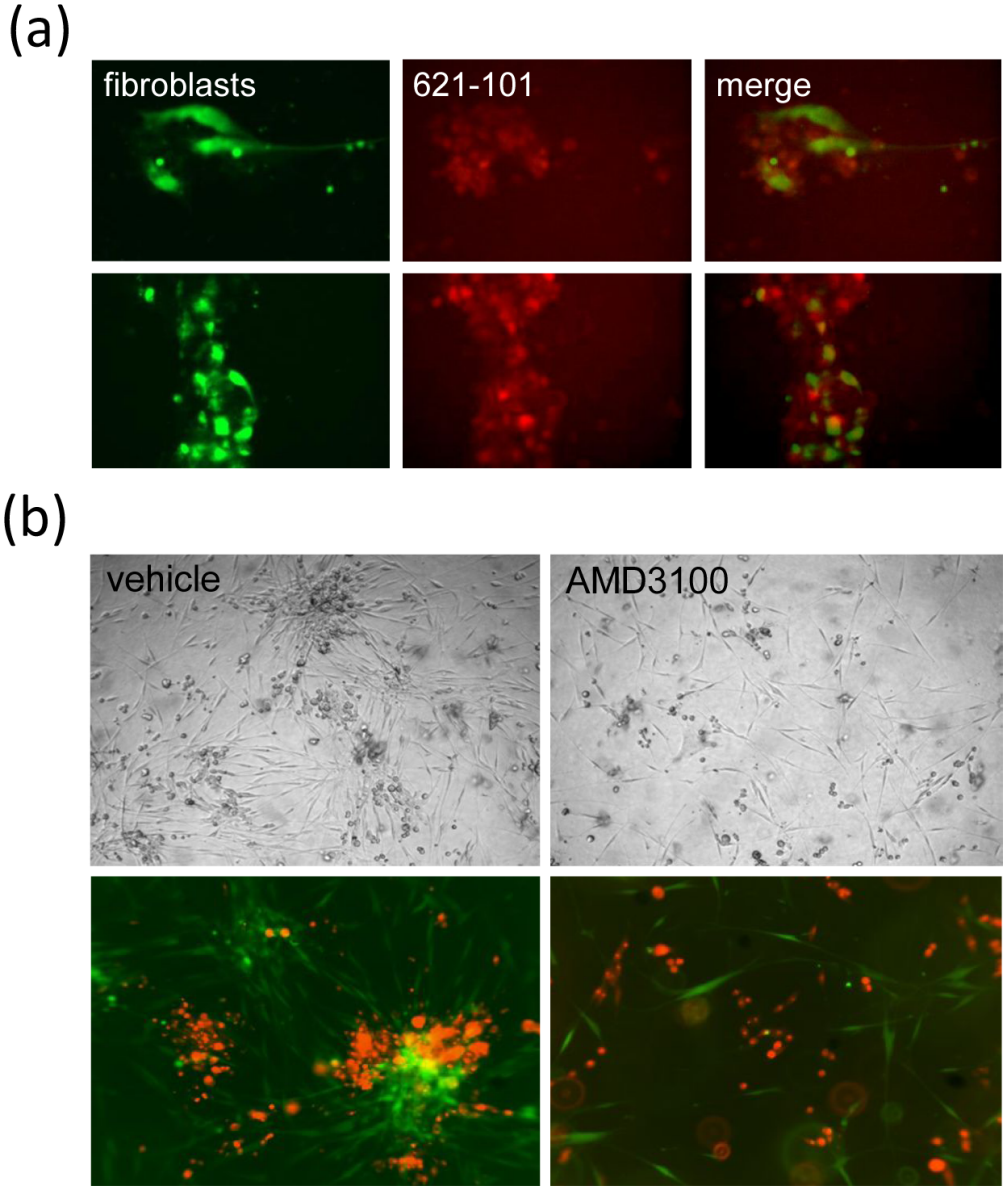
LAM 621–101 cells and fibroblasts form spontaneous aggregates in 3D co-cultures which are CXCR4/CXCL12 dependent. (a) 621–10 cells (red) and fibroblasts (green) are co-cultured in a three dimensional extra-cellular matrix gel and imaged by confocal microscopy. After three days cells spontaneously form clumps with fibroblasts appearing to surround 621–101 cells. (b) Addition of 200μg/ml AMD3100 results in fewer and smaller 621–101 cell—fibroblast aggregates.

We hypothesised that fibroblast-like cells are recruited to LAM cells by a mechanism akin to the recruitment of stromal cells by neoplastic cells in cancer. Examining this phenomenon in 3D extracellular matrix gels we observed that in both 621–101 cell and fibroblast 3D monocultures, cells remained dispersed diffusely throughout the gel over the course of the experiment. In co-cultures, after three days, spontaneous aggregates containing both cell types were observed, with fibroblast projections surrounding 621–101 cells (Fig 6a). To investigate the mechanism behind the spontaneous aggregation of fibroblast-like cells and LAM cells we examined the role of the CXCR4 / CXCL12 axis which is responsible for the recruitment of cancer associated fibroblasts in tumours. The addition of the CXCR4 receptor antagonist AMD3100 reduced the number and size of cell aggregates formed in this 3D LAM cell—fibroblast co-cultures (Fig 6b).

**Fig 7:**
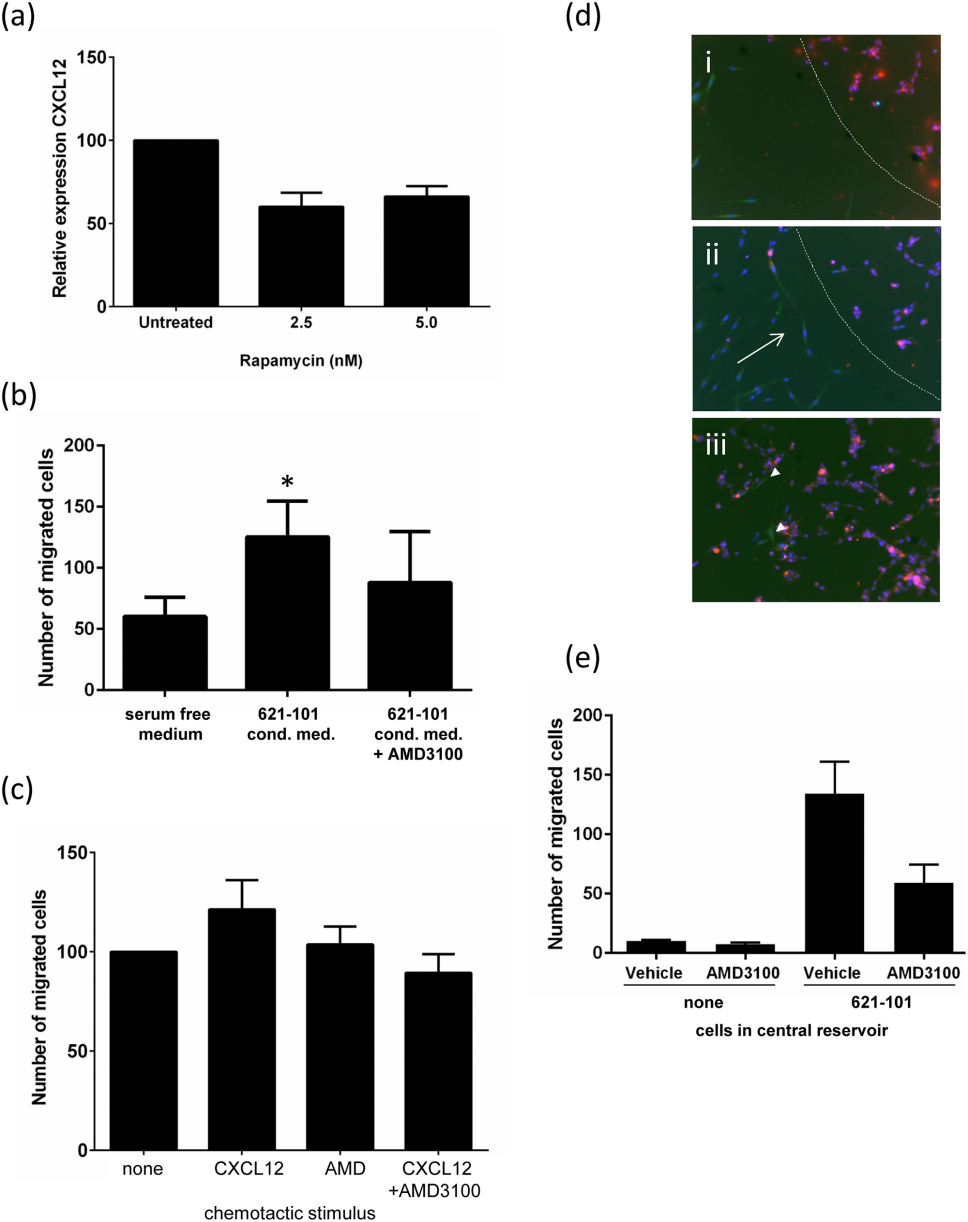
LAM cell medium is chemotactic for fibroblasts, and this is partially dependent on CXCR4. (a) 621–101 cells secrete CXCL12 protein; this is partially sensitive to rapamycin. (b) The presence of 621–101 cell conditioned medium (CM) increases fibroblast migration, which is partially blocked by AMD3100. (c) Fibroblasts migrate towards CXCL12 in a modified Boyden chamber (Transwell) assay; this is inhibited by the CXCR4 receptor antagonist AMD3100 at 100μg/ml (*p = 0.05). (d) Fibroblasts migrate towards 621–101 cells in a bidirectional migration assay. (i) Removal of the fence leaves a gap between the peripheral fibroblasts (green) and the central reservoir of (red) 621–101 cells (dotted), (ii) Fibroblasts migrate towards the central reservoir (arrowed), (iii) fibroblasts migrate into the central area (arrowed). (e) Addition of 200μg/ml AMD3100 reduces migration of fibroblasts in response to 621–101 cells by 56%. In the absence of 621–101 cells in the central reservoir migration is reduced by 93%.

In 2D cell cultures, 621–101 cells secreted CXCL12 into conditioned medium with unstimulated levels of CXCL12 expression in the range 250–300pg/ml. CXCL12 secretion was reduced 50% by rapamycin (5nM) (Fig 7a). In a Transwell migration assay, 621–101 cell conditioned medium resulted in a two fold increase in fibroblast chemotaxis (p = 0.015) over non-conditioned medium, which was almost completely abrogated by the CXCR4 antagonist AMD3100 (Fig 7b). Addition of recombinant CXCL12 resulted in a 21% (SD+/- 14%) increase in fibroblast chemotaxis (Fig 7c).

We additionally examined this phenomenon in a bidirectional migration assay which enables labelled fibroblasts and 621–101 cells to be plated in separate areas of tissue culture wells divided by a removable ‘fence’. Fibroblasts were placed in the peripheral section of the well, in the presence or absence of 621 cells in the central reservoir, in serum free medium. We noted that after 96 hours in culture human lung fibroblasts had migrated towards and into the 621–101 section of the well but no 621–101 cell migration towards the fibroblast area was observed (Fig 7d). There was significantly less migration of fibroblasts in the absence of 621 cells (p<0.05). Addition of 200μg/ml AMD3100 reduced migration of fibroblasts in response to 621–101 cells by 56% (Fig 7e).

### Protection from apoptosis

**Fig 8:**
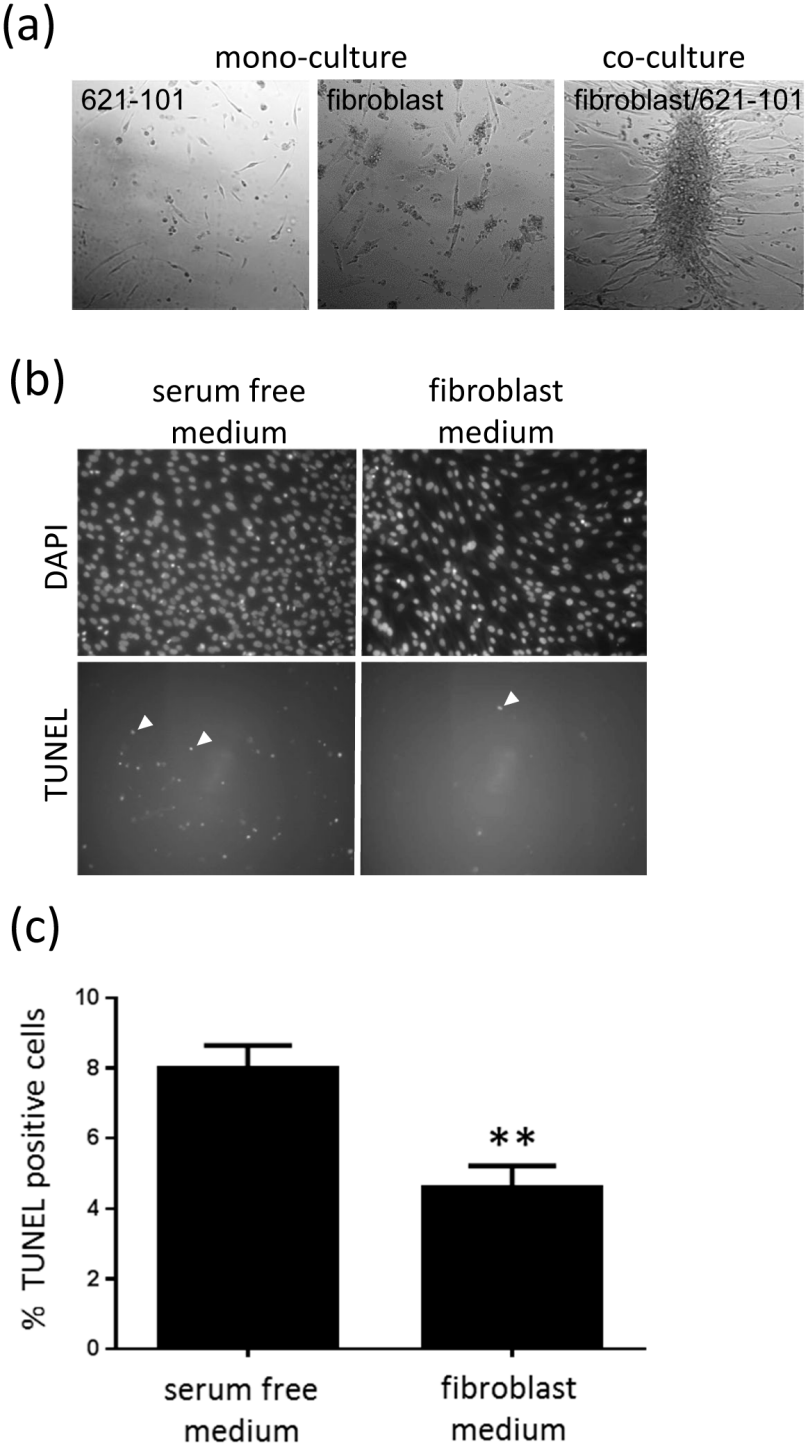
Fibroblasts protect 621–101 cells against apoptosis. (a) Culture of either 621–101 cells or fibroblasts on glass in serum free conditions results in cell death. Co-culture of the two cell types under the same conditions results in formation of viable cell colonies. (b) Under serum-free conditions, 621–101 cells have a basal rate of apoptosis of 8% assessed by TUNEL positive nuclear staining (arrowed). TUNEL positivity is reduced by half in the presence of fibroblast conditioned medium (*p = 0.032).

We observed that when either fibroblast-like cells or 621–101 cells were cultured independently in serum free conditions on low adhesion surfaces the majority of both cell types died over seven days. When fibroblast-like cells and 621–101 cells are grown together under the same conditions, cells remained viable and formed nodular aggregates (Fig 8a).

To determine whether the improved survival was mediated by a soluble factor we treated 621 cells with serum free medium on standard tissue culture plates. Under conditions of serum withdrawal 8% of the cells these cells undergo apoptosis, reflected by positive TUNEL staining (arrowed). If the serum free medium has been conditioned by prior incubation with fibroblasts for 24 hours, the proportion of apoptotic cells falls to 4.6% (p = 0.032. Fig 8b and 8c).

## Discussion

Our findings suggest that wild-type fibroblast-like cells can form a significant proportion of the cells within LAM lung nodules. These spindle-shaped cells express α-Smooth Muscle Actin, Fibroblast Surface Protein and Fibroblast Activation Protein but not markers of melanogenesis. Fibroblast-like cells harvested from LAM lung tissue have functional TSC-1/2 activity as they respond to serum withdrawal by suppression of mTORC1 signalling, unlike cells which have lost tuberin function in which S6 phosphorylation is maintained in the absence of serum. Our findings also suggest that LAM cells, by production of chemokines including CXCL12, promote the recruitment of these cells, and that their presence may confer a survival advantage.

We have used the expression of the melanoma markers gp100/PMEL and PNL2-antigen as an indicator of the presence of LAM cells. The presence of melanoma markers in lung is atypical and is used as a diagnostic indicator of LAM; it is likely that these cells carry the TSC2 mutation. We are, however, unaware of any research which directly correlates the presence of these markers in a cell with loss of TSC2. We also assume that the expression of these markers is a stable feature of LAM cells.

Our data are consistent with a model in which both LAM cells and fibroblasts are present in LAM lesions, but we cannot exclude the possibility that LAM cells expressing melanoma antigens lose these markers and adopt a less specialised state, involving expression of fibroblast markers. In this latter model, the LAM nodule would be clonal but comprise cells with different phenotypes. We favour the former model, as the low detection level of a TSC2 mutation or evidence of mTOR dysregulation in cells derived from LAM lung argues against the presence of large numbers of clonally derived mutation-carrying LAM cells.

Whilst some markers have been useful in the identification of fibroblasts in specific contexts, no marker is entirely specific for fibroblasts. α-Smooth Muscle Actin expression is the defining characteristic of activated fibroblasts (also called myofibroblasts), in which proliferation and the expression of ECM remodelling enzymes are increased relative to quiescent fibroblasts. Fibroblast Surface Protein (FSP) is an epitope of a surface protein found on primary cultured human fibroblasts [23, 24]. S100A4 is a marker of fibroblasts in tissues undergoing remodelling, for example in fibrosis in the renal interstitium [25], while FAP expression is associated with activated fibroblasts in tumour stroma and fibrotic disease [26]. We found expression of all of these markers in LAM tissue. The fibroblast-like population within a nodule does not appear to be homogeneous. S100A4 has more limited expression than other fibroblast markers and may identify a population of fibroblast like cells which has a distinct origin or phenotype from other (FSP and FAP-reactive cells). Given the lack of entirely specific markers for fibroblasts, however, we describe these cells as ‘fibroblast-like’, in that they display many of the markers used to detect fibroblasts in other diseases.

The expression of α-Smooth Muscle Actin and melanoma markers is used diagnostically to identify LAM cells in lung biopsies: however our use of a wider panel of markers suggests that α-Smooth Muscle Actin expression may be due both to the presence of LAM cells and fibroblasts. This, coupled with the presence of cells which do not express melanoma markers, including the recently described αPEP13h, a high sensitivity antibody targeting PMEL/gp100 [27], in LAM nodules suggest these lesions contain multiple cell types and suggest that cells similar to activated fibroblasts are present in LAM nodules, and are likely to contribute to the lung pathology of LAM.

Whilst we cannot be certain that the cells we recover from LAM lung tissue are the same cells that are immunoreactive to anti-fibroblast markers in paraffin sections: the cultured cells have the characteristic fibroblast spindle-shaped morphology and express the same markers which have proved useful in specific contexts for identifying fibroblasts. The recovery of fibroblasts in large numbers from LAM lungs may therefore be seen as confirmatory evidence for the presence of fibroblasts in LAM nodules as identified by immunohistochemistry.

LAM has many similarities to a neoplastic disease: LAM cells are clonal, disseminating, and TSC-2 null cells have a metabolic signature suggesting an increased reliance on glycolysis and upon glutamine as a substrate [28]. This link with cancer biology suggests that the recruited mesenchymal cells in LAM nodules may be analogous to cancer associated fibroblasts (CAFs). Indeed, CAFs characteristically have a signature characterised by expression of α-smooth muscle actin, MMP-2, PDGFR, TGFβ and FSP-1, all of which have been reported in LAM lesions. CAFs are a heterogeneous group of cells and derived from local fibroblast populations [29], bone marrow-derived mesenchymal stem cells and pericytes [30].

We have previously demonstrated CXCR4 expressing cells in LAM nodules, with more limited expression of its ligand CXCL12 [31]. The CXCR4-CXCL12 axis has been implicated in many aspects of cancer progression, including angiogenesis, metastasis, survival and homing [32]. CXCR4 is a target for novel cancer therapies and small molecule inhibitors of CXCR4, such as plerixafor (AMD3100), are being investigated clinically in various cancers [33]. CXCL12 is also a critical factor in the recruitment of fibroblasts by lung stem cells, generating a microenvironment which maintains stem cells in an undifferentiated proliferative state [34]. Our findings that fibroblast recruitment by 621–101 cells was at least partially dependent upon the CXCR4/CXCL12 axis in multiple assays suggest a role for this mechanism in LAM.

The association between tumour cells and CAFs is thought to be mutually beneficial supporting angiogenesis, metastatic behaviour and providing metabolic substrates. Our data suggest that the recruitment of fibroblasts also generates a microenvironment supportive to LAM cell growth and survival. As in cancer stroma, cross talk between (neoplastic) LAM cells and fibroblasts may result in a change in recruited fibroblast phenotype, such that their proteolytic activity is enhanced [35]; and we speculate that destruction of the lung parenchyma could be a secondary effect of fibroblast recruitment rather than a direct effect of TSC2^-/-^ LAM cells. Indeed, the contribution of fibroblasts (and perhaps other recruited cells) to the pathology of the LAM is currently unclear. Genotyping analyses suggest that the contribution of wild type cells to LAM tissue may be significant, to the extent that it can be challenging to detect the presence of a TSC-2 mutation at any level [15].

In conclusion, much research in LAM is focussed on the consequences of mTOR dysregulation in cells which harbour TSC2 mutations but which are not derived from LAM lung tissue. Our findings raise the possibility that only a minority of cells in a LAM lesion may be susceptible to mTOR inhibition and additional avenues for therapeutic intervention need to be explored, including targeting cell recruitment pathways, in order to fully address all aspects of disease progression.
